# P-417. Machine Learning Prediction of Pediatric Bacteremia: Development of EHR-Based Models for Diagnostic and Clinical Decision Support

**DOI:** 10.1093/ofid/ofaf695.634

**Published:** 2026-01-11

**Authors:** Nicholas P Marshall, Fatemeh Amrollahi, Fateme Nateghi Haredasht, Kameron Black, Aydin Zahedivash, Manoj Maddali, Stephen Ma, Amy Chang, Stan Deresinski, Mary Kane Goldstein, Steven Asch, Niaz Banaei, Jonathan H Chen

**Affiliations:** Stanford University, Palo Alto, CA; Stanford University, Palo Alto, CA; Stanford University, Palo Alto, CA; Stanford Medicine | Children's Health, Palo Alto, California; Stanford Medicine | Children's Health, Palo Alto, California; Stanford University, Palo Alto, CA; Stanford, Palo Alto, California; Stanford University, Palo Alto, CA; Stanford Health Care, Stanford, CA; Stanford University, Palo Alto, CA; Stanford University, Palo Alto, CA; Stanford University School of Medicine, Palo Alto, CA; Stanford University, Palo Alto, CA

## Abstract

**Background:**

Pediatric blood cultures are frequently ordered but have low positivity rates (< 4%) in emergency departments (EDs), highlighting the need for better-targeted testing. Accurate prediction can reduce unnecessary cultures, conserve resources, and support stewardship—particularly during the global blood culture bottle shortage. Models developed for adults perform poorly in children due to physiological and clinical differences; in prior work, applying an adult model to pediatric data yielded an AUC of 0.61. We excluded infants < 90 days, who have distinct risk factors (e.g., perinatal history), and developed machine learning models to predict bacteremia in children aged > 90 days to ≤ 18 years using electronic health record (EHR) data.

**Table 1:**
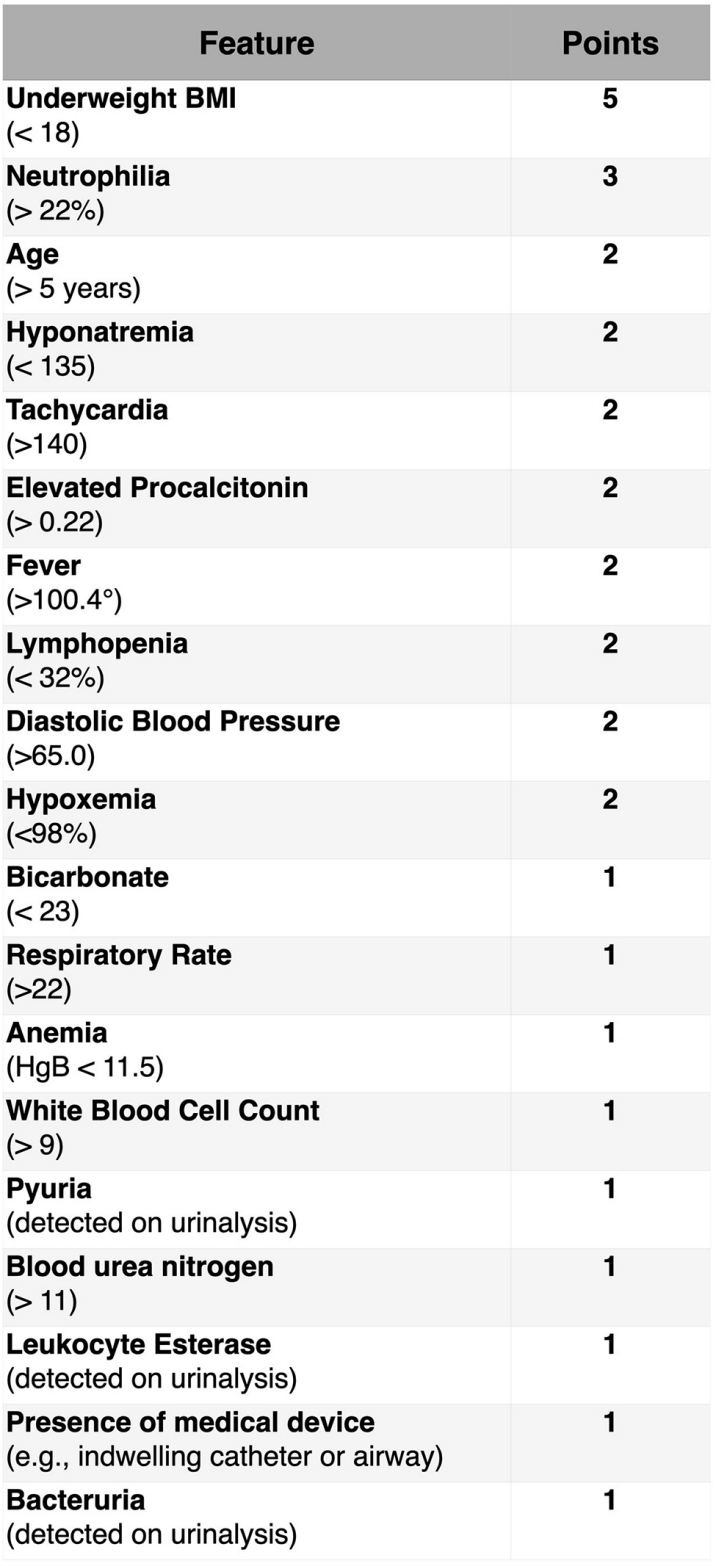
PedsBactoScore Point-Based Scoring System Derived from Logistic Regression Coefficients Each feature contributes a fixed number of points based on clinically meaningful thresholds. The total score is used to stratify risk of bacteremia at the point-of-care.

**Table 2:**
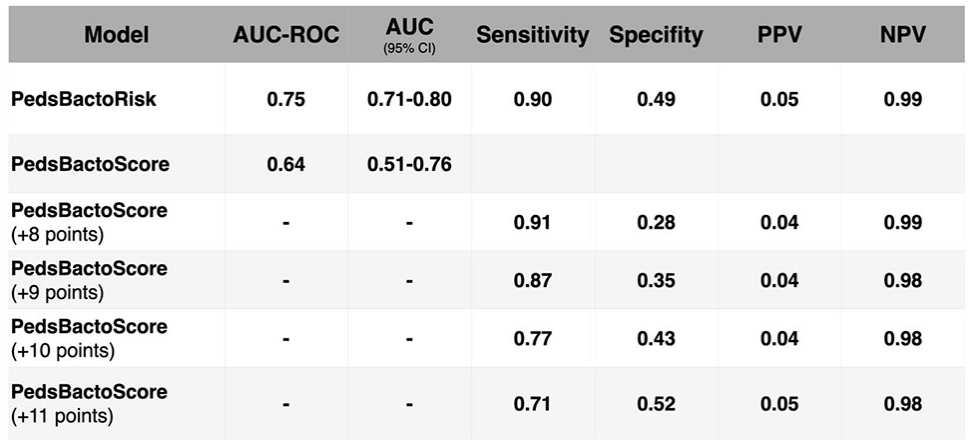
Performance Metrics of Pediatric Bacteremia Prediction Models Comparison of PedsBactoRisk and PedsBactoScore models on the pediatric test set. Metrics include AUC with 95% confidence intervals, sensitivity, specificity, positive predictive value (PPV), and negative predictive value (NPV) at pre-specified thresholds.

**Methods:**

We analyzed 26,829 blood culture orders from 9,362 pediatric emergency department (ED) encounters at Stanford Medicine | Children's Health. To preserve temporal validity, data were split chronologically, with the most recent encounters used as the test set. We developed two models: PedsBactoRisk, a logistic regression model, and PedsBactoScore, a simplified point-based tool derived from the most influential PedsBactoRisk predictors. The PedsBactoScore rubric is shown in Table 1.

**Results:**

Table 2 summarizes model performance. We evaluated sensitivity, specificity, PPV, and NPV, focusing on thresholds achieving 90% sensitivity. PedsBactoRisk achieved an AUC-ROC of 0.75; PedsBactoScore, 0.64. While PedsBactoRisk showed superior performance, PedsBactoScore allows easier implementation via its interpretable scoring system. PedsBactoScore performance is shown across thresholds to illustrate sensitivity–specificity trade-offs.

**Conclusion:**

PedsBactoRisk demonstrated the highest overall performance (AUC: 0.75), but PedsBactoScore offers a pragmatic, interpretable bedside tool with strong sensitivity. Both models support more judicious blood culture use by identifying low-risk patients with high sensitivity. Future work will focus on integrating provider notes using large language models to enhance predictive accuracy and extending this approach to infants < 90 days by incorporating maternal and delivery data.

**Disclosures:**

Jonathan H. Chen, MD, PhD, Reaction Explorer: Ownership Interest

